# Involving traditional birth attendants in emergency obstetric care in Tanzania: policy implications of a study of their knowledge and practices in Kigoma Rural District

**DOI:** 10.1186/1475-9276-12-83

**Published:** 2013-10-14

**Authors:** Dismas B Vyagusa, Godfrey M Mubyazi, Melchiory Masatu

**Affiliations:** 1 Singida District Council, P.O Box 354, Singida, Singida Region, Tanzania; 2 National Institute for Medical Research (NIMR), Headquarters, 2448 Barak Obama/Luthuli Road (former Luthuli/Ocean Road), P.O Box 9653, Dar es Salaam, Tanzania; 3 Centre for Educational Development in Health, P.O. Box 1162, Arusha, Tanzania

**Keywords:** Obstetric care, Pregnancy, Traditional birth attendants, Maternal health, Tanzania

## Abstract

**Introduction:**

Access to quality maternal health services mainly depends on existing policies, regulations, skills, knowledge, perceptions, and economic power and motivation of service givers and target users. Critics question policy recommending involvement of traditional birth attendants (TBAs) in emergency obstetric care (EmoC) services in developing countries.

**Objectives:**

This paper reports about knowledge and practices of TBAs on EmoC in Kigoma Rural District, Tanzania and discusses policy implications on involving TBAs in maternal health services.

**Methods:**

157 TBAs were identified from several villages in 2005, interviewed and observed on their knowledge and practice in relation to EmoC. Quantitative and qualitative techniques were used for data collection and analysis depending on the nature of the information required.

**Findings:**

Among all 157 TBAs approached, 57.3% were aged 50+ years while 50% had no formal education. Assisting mothers to deliver without taking their full pregnancy history was confessed by 11% of all respondents. Having been attending pregnant women with complications was experienced by 71.2% of all respondents. Only 58% expressed adequate knowledge on symptoms and signs of pregnancy complications. Lack of knowledge on possible risk of HIV infections while assisting childbirth without taking protective gears was claimed by 5.7% of the respondents. Sharing the same pair of gloves between successful deliveries was reported to be a common practice by 21.1% of the respondents. Use of unsafe delivery materials including local herbs and pieces of cloth for protecting themselves against HIV infections was reported as being commonly practiced among 27.6% of the respondents. Vaginal examination before and during delivery was done by only a few respondents.

**Conclusion:**

TBAs in Tanzania are still consulted by people living in underserved areas. Unfortunately, TBAs’ inadequate knowledge on EmOC issues seems to have contributed to the rising concerns about their competence to deliver the recommended maternal services. Thus, the authorities seeming to recognize and promote TBAs should provide support to TBAs in relation to necessary training and giving them essential working facilities, routine supportive supervision and rewarding those seeming to comply with the standard guidelines for delivering EmoC services.

## Background

Until today in developing countries high maternal mortality ratios (MMR), infant mortality ratios (IMR), high number of stillbirths, and abortions prevail. These together with the disabilities facing mothers during pregnancy or after delivery and problems facing the newborn babies as a result of the pregnancy related complications and those featuring during childbirth are one of major public health policy challenges [1,2]. According to evidence available, the main causes of maternal mortality in many of these countries are haemorrhage, infections, pre-eclampsia and eclapsia, obstructed labour and unsafe abortion. However, the factors contributing to these conditions are diverse [3-5]. Generally, the reports continue revealing that a considerable number of pregnant women in the least income countries (LICs) die unattended by skilled service givers and the deaths normally occur in many forms due to various causes. Some of the death events occur while the mothers concerned are on their way toward seeking skilled pregnant care at formal health care facilities or while delivering at home in the absence of skilled attendants [6,7]. At times, the delivering women in remote rural settings are forced to be attended by other people such as their in-laws, neighbours, other elderly women or by traditional birth attendants (TBAs), leave alone those who die at health facility levels after failing to be attended immediately by skilled workers [7,8]. TBAs have been defined by the World Health Organization (WHO) Expert Committee as persons who assist mothers during childbirth, and these women have initially acquired some skills by participating themselves in assisting mothers to deliver babies or through apprenticeship to other TBAs [7].

Proponents have been arguing that if TBAs had the appropriate skills required, they could deliver appropriate care to save lives of many women living in hard to reach settings. Such proponents or advocates maintain that sometimes pregnant women attending antenatal care (ANC) clinics late sometimes because of their negligence, but sometimes due to such factors as unaffordable travel costs. Apart from travel related costs, pregnant women sometimes do experience while at health facility levels unaffordable or unacceptable user fees (official or unofficial), disappointing long waiting time for service, shortages of service (e.g. drug), unfriendly handling by service givers, and generally low trust in the health care services available at formal health care facilities. Moreover, a considerable number of women find themselves being faced with domestic occupational commitments that keep them too busy for them to secure convenient time for visiting health care facilities [9-13]. In bushy areas, some women decide not to contact formal health facilities in fear of possible attacks from wild animals such as lions [14-16]. Furthermore, evidence shows that in Africa and some Asian countries, community recognition of TBAs’ services is very high and therefore TBAs are highly respected and consulted by the local people including pregnant women on various pregnancy health related matters. One of the reasons for this happening is that TBAs live close to the communities among which there are people who need their service. As time goes on, TBAs eventually become known and appreciated for their service and become more familiarized with their local clients than the health facility-based skilled personnel. Some of the latter personnel are non-indigenous in their duty stations and therefore continue being seen as foreigners or outsiders. TBAs are also often consulted because they often do not claim monetary payments from their clients for the given services [8,17]. The question that has prevailed in the maternal health research and policy literature relates to TBAs’ ability to deliver appropriate services, including those relating to emergence obstetric care (EmoC). WHO’s and other experts’ reports reveal that EmoC is essential for saving the lives of pregnant women during labour and their babies after childbirth [18,19].

Analysts of maternal deaths occurrence have also critically discussed the issue of the timing of such deaths in various situations and countries. Globally, records show that most of the deaths facing pregnant women that occur at home are recorded during the first postnatal or postpartum week, and particularly within the first 24 hours of birth. Such deaths are mostly caused by unsafe abortions [20-22]. Meanwhile, the issue of poor quality of care at formal health facilities continues to be observed as a contributing factor to maternal deaths in LICs. It is evident that in the countries of sub-Sahara Africa (SSA), the low quality of EmoC services at formal health facilities disappoints the women who are eligible or desiring to receive EmoC services. The tracer elements of the low services are reported to include such things as the shortages of skilled and motivated health workers, shortages of essential medicines and other important medical supplies including delivery kits and equipments as well as limited physical infrastructures. Meanwhile, there has been a general lack of acceptable referral facilities and system to meet the needs of clients in emergence conditions. As a result, public’s trust in the health-care systems and their morale for utilizing the available services have been greatly lowered, leading to underutilization of the available services, hence unnecessary deaths [23-27].

Tanzania is among the LICs with training programs relating to EmoC delivery and management issues. The training programs target to involve TBAs and are considered to be an integral part of the formal health service system [28]. Nevertheless, critics have continued questioning about the ability of TBAs to deliver safe pregnancy and childbirth services. The doubting individuals have been reporting to know on some of TBAs who have been illiterate and unable to perform their duties as recommended while they have continued delivering pregnancy and delivery services in poorly equipped and unsafe environments [29,30]. The general feeling or presumption among such critics is that even when the TBAs training programs have attempted to cover the important safe motherhood issues, the form of training given has often been inadequate when looking at the contents and curriculum coverage. Such critics continued depicting that there is a paucity of evidence on the ability of TBAs in meeting the EmoC service delivery conditions after attending training [31]. This concern prevails even today although evidence from several south Asian countries reveals that the use of trained TBAs has significantly saved the lives of pregnant women and the babies [32]. Meanwhile, it has been noted that whether or not TBAs are important in EmOC services, their availability, accessibility, competence and skills do vary widely across settings [33,34], leave alone the numerous reports from different countries also revealing that the health systems of such countries are weak by generally lacking effective supervision and monitoring of the trained TBAs [7,35]. Moreover, it is noted that even when TBAs are trained, their ability to adopt the required improved practices is not universal and the extra confidence they gain after being trained sometimes lead to higher incidences of dangerous procedures and sometimes delays in referring the women for specialized EmOC services [36-39].

In a nutshell, there has been a shift to policy debates identifying the need for the formal authorities concerned to replace TBAs with skilled birth attendants. In contrast, the opponents to this i.e. TBAs proponents have called for the maintenance of synergy whereby the formal health care provision system could recognize and work in cooperation with TBAs [40-42].

In Tanzania, reports on MMR show that the country has not achieved much in reducing the maternal deaths. However, such reports have tended to differ on the issue of the actual MMR recorded in different years. This is due to different sources of data and type of the data reporters [1]. The latest records obtained from the National Demographic and Health Survey (DHS) indicates MMR to be 454 per 100,000 live births [43]. As in many other countries within and outside of SSA, late and irregular pregnant women’s contact of ANC clinics and health facilities for ordinary services as well as shortage of skilled health personnel and basic supplies at health facility levels are noted to be the contributing factors [44-46]. In Tanzania, less than 50% in urban areas and less than 40% of their counterparts in rural areas have been delivering in health facilities under help from skilled personnel [2,47]. Use of TBAs has remains to be a common practice in this country. Previous reports estimated Tanzania to possess about one million traditional practitioners, and among these 40% were TBAs [48]. This proportion is large enough to cover a significant proportion of the women who under situations of limited services or facilities reported above cannot endure visiting health facilities where they have no hope of getting the desired help. Given the current conflicting evidence from studies carried out so far, the ability of TBAs in dealing with EmOC in Tanzania is still a major research and policy questioned.

With the intention of collecting more evidence toward answering the outstanding questions, the present paper reports a study that was designed and carried out based on a review of past studies on TBA issues in Tanzanian districts, focus being Kigoma Rural District. At the time of the present study conception, reports from a study by Mbaruku and others [49] showed that the Kigoma Rural District recorded the MMR of 757 per 100,000 live births and this rate was higher than the rates recorded in the rest of the districts in the same region of Kigoma. Following the latter study’s findings and recommendations, among which was the need for using trained TBAs if they were to perform as desired, the authority of Kigoma Rural District Council decided to train 160 TBAs between the years 1999 and 2001. The trained TBAs were prepared to serve better in safe motherhood service programme. It was also recommended to monitor and evaluate the performance of these trained TBAs. That is why the present study came to be important as one way of evaluating the role of this training programme and finding out additional evidence on the ability and roles of TBAs. One of the objectives of this study was to explore the knowledge of the trained TBAs on EmoC services and their practices. The investigation focused on, among other things, how the study participants were complying with the guidelines for the delivering the required services. Observations were also done to establish whether or not TBAs’ working environment was suitable for the safe and appropriate care to be delivered to the clients as recommended. The study was conducted in a similar manner as the one done in Ghana to evaluate the extent to which trained TBAs remembered what they had been taught at the training sessions three years ago [50].

## Materials and methods

### Study design

The study adopted a cross-sectional survey design. Being descriptive in nature, it involved the gathering of qualitative data by interviewing TBAs at the places which they preferred to be interviewed. This was supplemented with observations of TBAs’ practices. Quantitative data were also gathered as part of interviews done with TBAs. As mentioned above, the main study theme was about TBAs’ knowledge about EmoC aspects and their practices in delivering the desired services to the women in need. Other aspects investigated are as described below under the data collection section.

### Study areas and population

The study covered 6 divisions, namely Kalinzi, Mahembe, Mwandiga, Nguruka, Buhingu and Ilagala, and all of them are located in Kigoma Rural District. This district is found between latitudes 4–6 degrees South and longitudes 29–30 degrees East. The district was selected based on the report indicating the district to have recorded a MMR of 757 deaths per 100,000 live births, the rate that was higher than the rates reported from elsewhere in Kigoma Region [49]. The latter Region is situated on the western border of Tanzania and has four administrative districts, namely Kigoma Urban (Kigoma Ujiji), Kibondo, Kigoma Rural (R) and Kasulu. The main portion of Kigoma (R) district lies along the shore of Lake Tanganyika whereby about 50% of her population lives. The Kigoma (R) district was found to be occupied with poor roads and other communication network systems including telephone infrastructure. According to the reports from the health authorities of this district and the experience of the first author in the present paper, communication was a challenge when it came to getting immediate information related to patients needing referral services. The majority of its inhabitants belonged to the 'Ha’ tribe and Ha is the most popular indigenous language. Most of the residents were found being small-scale farmers, growing such cash crops as coffee and palm while potatoes, cassava, beans, maize and bananas were being grown mainly for food. Until 2005 when this study was being undertaken, there was neither any government (public) hospital nor any private hospital and therefore all referral and advanced services were being sought at the regional hospital known as Mawenzi while a few cases were referred to the private Baptist Hospital. Both of the latter two hospitals are situated in Kigoma Urban district. In terms of total number of health facilities and their types, the Kigoma (R) district possessed 5 public health centres (only two of these belonged to voluntary agencies while the rest were publicly (government) owned)). Until 23^rd^ March 2013, records showed that the district had over 63 dispensaries among which 2 belong to parastatal organizations, 2 owned by Faith Based Organizations (Voluntary Agencies) and 2 belonging to private-for-profit entities (District Council Health Authorities, Kigoma Rural, per comm.). As some of these facilities were not easily accessible most of the deliveries were home-based.

### Sampling

Originally, the plan was to recruit 160 trained TBAs among those who were registered by the District Council Health Authority. But, only 157 could be reached until end of the study. This depended on several factors especially the presence of the individuals targeted. Those who eventually were mobilized to participate have been selected using a convenience sampling approach. There was no baseline statistics on the total number of TBAs who were registered and those who were not registered so as to be used for calculating in advance of the study the sample representing different localities and gender mix for reasonable representation. The TBAs who were at last reached were found in all 6 divisions of the district and were identified through Consultation with local community leaders and community health workers.

### Data collection

As highlighted before, qualitative and quantitative data collection techniques were adopted. This involved the gathering of the data using the data collectors after being oriented on the proper procedures. The data collectors were familiarized with both the use of research instruments, seeking informed consent from the study participants and carrying out field observations. The consent sought was either supported by each participant’s signature or thumb print as further elaborated later. Among these collectors were members of district health council management team (CHMT) and a few health facility based staff who were routinely working on maternal and child (MCH) services at the ANC and child health clinics. The staff concerned includes health officers, clinical officers and people with nursing profession. These were included in order to enable them see themselves the working environment of TBA in their delivery places, knowledge of TBAs on EmOC aspects, and their suggestions in relation to how the prevailing MCH service problems could be addressed if TBAs were to continue being involved effectively. This was believed to be an opportunity for equipping the staff concerned with the practical hands on answers about the working ability and environment of TBAs, thus enhancing their chance to recognize how better the experience gained from the field could be utilized for better actions. The individuals concerned were also expected to give the appropriate advice to any authorities that were much concerned with issues relating to TBAs’ involvement in maternal health services. This includes consideration on all possible or better practical ways of working together with TBAs in the respective communities. Careful measures were taken to ensure that the health service personnel involved in the data gathering process were those who were not popular or known. in the respective study communities. This was aimed at minimizing if not to avoiding obvious study biases or some respondents refraining from providing some key information. However, in a way, a few of the staff were found to be known in the study settings.

Data were collected between April and May in 2005. In general, the information collected using different data collection tools (questionnaires, observational checklist, etc.) was based on questions intended to establish the respondents’ knowledge about EmoC issues and their practices in delivering the services to the needy pregnant women. A questionnaire used was translated in Kiswahili for easier use during the interviews with the identified TBAs. It comprised of a mixture of closed-ended and open ended questions. The former type of the questions was aimed at obtaining quantitative data that were supplemented with the data gathered using open-ended questions. The latter questions were mainly aimed at coming up with qualitative data that would supplement or help to explain some statistical data. The specific issues upon which the study questions were based addressed the respondents’ knowledge about the signs and symptoms of pregnant women who were at risk of facing maternal complications and risk of contracting HIV/AIDS infections. The respondents were also examined about their knowledge on the modes of HIV/AIDS transmission and prevention, the referral procedures, obstetric handling procedures for the pregnant women during labour as well as during and after childbirth (e.g. by observing aseptic techniques). Other issues investigated related to measures taken by TBAs after encountering a woman with haemorrhage and/or those who were facing other problems before, during and after childbirth. The interview schedules were conducted in one of the rooms, and this was either at the nearby study health facilities or in the hamlet leaders’ houses in the visited study villages. It was in such villages from where TBAs recruited for study were also selected. The places identified for interview have been selected after consultation with the TBAs themselves who felt that they could be more conveniently investigated while being there than and elsewhere. Their choice was respected instead of the previous plan of conducting the interviews and observations in the participants’ own premises. It was discovered that for various reasons the TBAs could not be comfortable if the study team saw the places where they were delivering their services.

Moreover, field observations were conducted on TBAs’ delivery kits, interest being on the items contained in the kits, kit completeness and cleanness. Other planned issues for observation include TBAs’ service delivery environments, particularly state of the buildings and general surroundings in terms of sanitation and hygiene. Moreover, part of the observation include a review of TBAs’ note-books and the interest behind was to obtain the records showing whether or not the referrals were being made in correct way. To facilitate both the interviews and observations, TBAs were asked in advance to come with their delivery kits on the day they participated in the study.

### Data management and analysis

Each day after fieldwork, the research team was meeting to review and discuss the data collected by checking for their completeness and inconsistencies, making necessary corrections and compiling the data. Where applicable, the responses to open ended questions appearing on the questionnaire were coded to allow their frequency tabulation. The coded data were double entered in the database prepared using EPI-Info program. Analysis of these structured data was performed using SPSS software package. The one-way frequency tabulations were followed by chi-square (χ2) test technique so as to assess if there were any statistically significant difference in the observations made with respect to on the computed variable proportions. The difference observed was considered to be statistically significant at a P ≤ 0.05.

To understand the level of knowledge among the respondents on signs, symptoms and danger signs of pregnant women at risk, a scale was used whereby respondents who mentioned at least three conditions were counted 'highly or adequately knowledgeable’. Those who mentioned only one-to-two conditions were counted 'moderately knowledgeable’ while those who could not mention any condition were categorized 'poorly knowledgeable’. The analysis was thus limited in that no logistic regression analysis was performed. This is because it was found that the analysis done was still sufficient to help the study objectives to be achieved given the nature of the data collected. As shown under the results section, the denominators used in the calculation of the frequencies presented in the results section varied from one question to another and this was due to how the interviewees responded to specific questions. The unmentioned answer options or missing variables were excluded in the analysis.

### Ethical considerations

Official ethical clearance for the study was obtained from the Tumaini University through the Kilimanjaro Christian Medical Centre (KCMC) Ethics Committee. Permission to conduct the study in the district was also sought from Regional and District Medical and Administrative Offices. The study participants were informed about the study with assistance by local leaders. The individuals approached were also given the right explanations about the objectives and expected benefits of the study. They were also informed about the chance that anyone would be allowed to participate willingly and voluntarily. Those who were unable to read (and or write) were asked to put a thumb print on the informed consent form. The rest signed the consent form voluntarily. Participants were assured of the anonymity of their names and confidentiality of the information they wished to be treated so. All of the individual participants were informed of their freedom to drop out of the study any time they wished even after participating.

## Results

### Quantitative findings

#### Characteristics of the participants

About ninety eight percent 98.13% of the interviewees (N = 157) participated in the study. Of all the interviewees, 57.3% (n = 90) were in the age of 50 years or above, and nearly 50% had not received any formal education. Only 45% of the respondents reported to have received primary level education and these include those who attained at least standard seven. Other socio-demographic characteristics of the respondents are as indicated (Table 1).

**Table 1:** Socio demographic characteristics of the study participants

**Characteristic**	**n (%)**
**Age**	
20-29	1 (0.6)
30-39	26 (16.6)
40-39	40 (25.5)
50+	90 (57.3)
**Education**	
No formal education	78 (49.7)
Adult education	9 (5.7%)
Primary school	70 (44.6)
**Marital status**	
Married	113 (72.0)
Divorced	8 (5.1)
Widowed	35 (22.3)
Separated	1 (0.6)
**Religion**	
Muslim	70 (44.6)
Christian	87 (55.4)

#### Knowledge on danger signs during pregnancy

With regard to whether the TBAs were taking their clients’ history of the pregnancy conditions before giving the service, 89.7% (n = 140) who answered the question reported to have been checking their clients’ ANC cards regularly before deciding to give the service. The reporters were aware that on those cards there was a section whereby the client’s personal pregnancy history is usually recorded at health facility level and that these were the ones being checked on the issue of pregnancy gestational age and danger/risk conditions if any. Only one interviewee did not say anything on this issue for unexplained reasons. The rest reported to have had never seen an ANC card at all. Occasional meeting of pregnant women carrying their ANC cards with them when visiting TBAs was affirmed by 61.5%, (n = 96) of the respondents; the rest denied to have ever met the clients coming with such cards.

As for those who knew about the danger signs of pregnancy, 94.7% (n = 108) affirmed to have at least heard about such signs before. However, only 31.9% (n = 47) were able to pinpointed abnormal lying of the baby or big baby; 52.7% (n = 78) mentioned hemorrhage, low hemoglobin or retained placenta; whereas 15.5% (n = 23) identified such other conditions as abnormal presentations, fits during pregnancy, oedema or weaknesses. There was not statistically significant difference observed as existing in terms of knowledge of the said conditions between the respondents who were found to be relatively more educated (those who completed at least standard seven primary education) and those who never attended primary school at all. The same observation was made by comparing the TBAs who were found to have been practicing in rural settings and their counterparts who were practicing in semi-urban settings. When the respondents were asked about whether or not the pregnant women consulting TBAs who were respondents to this study presented the signs of pregnancy risks, the answers indicating 'Yes’ were obtained from 111 interviewees. Among these, 58.9% (n = 65) expressed adequate/high knowledge of the appropriate risk signs; 17.1% (n = 19) expressed moderate knowledge; while 9.0% (n = 10) had poor knowledge.

#### Awareness and knowledge on HIV/AIDS transmission and prevention

All of 157 respondents were found being aware that HIV/AIDS was a life-threatening and yet an incurable disease and that it was one of the major public health problems in the community. Among these interviewees, 92.4% (n = 145) knew that HIV could be transmitted through sexual intercourses or coming into skin contact with the blood of an infected person; 5.7% (n = 9) did not know the modes of HIV/AIDS transmission while 1.9% (n = 3) mentioned other unreal modes of transmission including the shaking of hands, eating together by sharing the same utensils e.g. plate or playing together, with the people infected with the HIV. The question asked to establish respondents’ knowledge about the recommended means of preventing HIV infections or transmission during service delivery was answered by 155 interviewees. Among these, 91.6% (n = 142) acknowledged to have been wearing gloves while attending the women delivering babies, 2.6% (n = 4) reported that they were just covering their hands with a piece of cloth, while 0.6% (n = 1) applied local herbs on her hands before assisting delivery. The respondents reporting not to know what to do accounted to 5.2% (n = 8). In this case also, no statistically significant difference was observed in terms of the responses given between the respondents who were found to have primary education and those who had not gone to school at all regarding the means of protection actually used. However, when asked about whether or not gloves were useful or necessary for helping the service providers to prevent themselves from contracting HIV infections while attending the delivering women, 99.4% (n = 155) responded affirmatively and only 0.6% (n = 1) felt that the use of gloves was unnecessary. As for how frequently one used the same pair of gloves between successive delivery/childbirth assistance processes before discarding/disposing them as waste materials, the answers were obtained from 152 interviewees and among these, 69.1% (n = 105) claimed to have been using one pair once; 21.1% (n = 32) used a pair twice, 7.2% (n = 15) used a pair three or more times; while the remaining (2.6%) could not even count the number of times they were using such materials. It was claimed by the respondents that as the gloves were more frequently used than expected, experience with shortages in the supply of such materials could not be avoided. Participants expressed concern with lack of support from the formal health system in relation to the supply of essential working gears and the poverty nature facing TBAs and/or their clients who come empty handed for delivery.

#### Management of bleeding occurring during pregnancy

Answers to the question on how the bleeding women during pregnancy were being managed were obtained from 157 of the interviewees. Only 42.2% (n = 71) of the respondents affirmed to have met such cases. Also, 87.3% (n = 62) said that they have been giving medicines; 8.4% (n = 6) said that they were referring their clients to formal health facilities, while 4.3% (n = 3) were just praying God for help without them administering anything.

#### Observing aseptic techniques during delivery and treating instruments after delivery

Responses to the question on how the TBAs were getting prepared to assist mothers during baby delivery were obtained in the following forms: washing hands - 19.1% (n = 30); both washing hands and wearing gloves - 49.1% (n = 77); wearing gloves without washing hands - 21% (n = 33); using nothing - 3.2% (n = 5), while those who were just praying God for help accounted to 7.6% (n = 12).

With regard to how the materials used for managing the pregnant women during childbirth were being handled after use, clear responses were obtained from 156 respondents and these were as follows: washing and then boiling them - 58% (n = 91) just boiling without washing them - 5.1% (n = 8); washing them only - 29.3% (n = 46); and keeping them unwashed - 7.6% (n = 12). It was interesting that 99.4% (n = 155) out of 156 respondents (as 1 person did not respond) viewed that the use of gloves when conducting a delivery service is necessary for preventing infections while one person (0.6%) said gloves were not necessary.

On the issue of the frequency at which vaginal examination was being conducted by the respondents to the pregnant women they were confronting, the answers obtained from all of the 157 interviewees indicated the following flow: examining their clients less than four times per each delivery - 91.6% (n = 144); never counted how many times examination was being done - 6.5%; examined more than five times per delivery - 1.3%; and never examine at all – 1 respondent. Stating the equipments used when assisting delivery, the answers obtained were as follows: 93.6% (n = 147) - use gloves, new razor blades, mackintosh and towel while the rest were only using scissors and antiseptics.

As for what the measures were being taken at in event of shortage of delivery equipments, clean answers were obtained from 156 interviewees and these were as follows: never participate in assisting mothers to deliver - 49.3% (n = 77); use any means available (including cloth, leaves, etc.) - 27.6% (n = 43); assisting without using any means of protection - 23.1% (n = 36).

#### Managing problems encountered before, during, and after assisting delivery

Responses to the question about the actions they have been taking whenever they found themselves being unable to help mothers to deliver safely indicated that: the majority, that is, 98% of the respondents reported that they were referring their clients to formal health facilities. As per the answers given, it seems that the referrals made were as follow: women with prolonged labour – 82.7%; mothers seeming too physically exhausted – 49%. Other problems either referred or not to health facilities are as listed in (Table 2). Also, 46% of the responses indicated that the pregnant woman concerned was allowed to rest and given food.

**Table 2:** Distributions of respondents on managements of problems during labor

**Problems**	**Services**	**n (%)**
*Prolonged labor*	Refer to hospital/dispensary	129 (82)
	Medicine	22 (14)
	Pray only	2 (1.3)
	Drinks	2 (1.3)
	Watching only	1 (0.6)
	Did not respond	1 (0.6)
*Exhausted mother*	Rest and food	72 (45.9)
	Refer to hospital/dispensary	77 (49.0)
	Others (Pray, medicine)	8 (5.1)
*Hand presentation by baby*	Deliver her	36 (22.9)
	Refer to hospital/dispensary	113 (72.0)
	Medicine	5 (3.2)
	Never happened	3 (1.9)
*Breech*	Deliver	79 (50.3)
	Refer to hospital/dispensary	76 (48.4)
	Medicine	2 (1.3)
*Cord presentation by baby*	Refer to hospital/dispensary	134 (85.4)
	Deliver	17 (10.8)
	Medicine	4 (2.5)
	Never happened	2 (1.3)
*Cord around neck of baby*	Deliver	129 (82.2)
	Medicine	6 (3.8)
	Refer to hosp/dispensary	20 (12.7)
	Did not respond	2 (1.3)
*Intra uterine fetal death*	Refer to hosp/dispensary	129 (86.6)
	Deliver	18 (12.1)
	Medicine	2 (1.3)

Experience of having been meeting with the mothers presenting anemia and retained placenta was shared by some of the respondents as follows: Yes, meet them - 22.8% (n = 29); Yes –experienced the mothers with abnormal lying of the baby, and mothers who fail to deliver. The latter was reported to be due to such mothers’ babies found to be too big or the mothers being grand multipara - 29.1% (n = 37).

#### Availability and use of delivery kits for TBAs, delivery environment and suggestions

**Figure 1:**
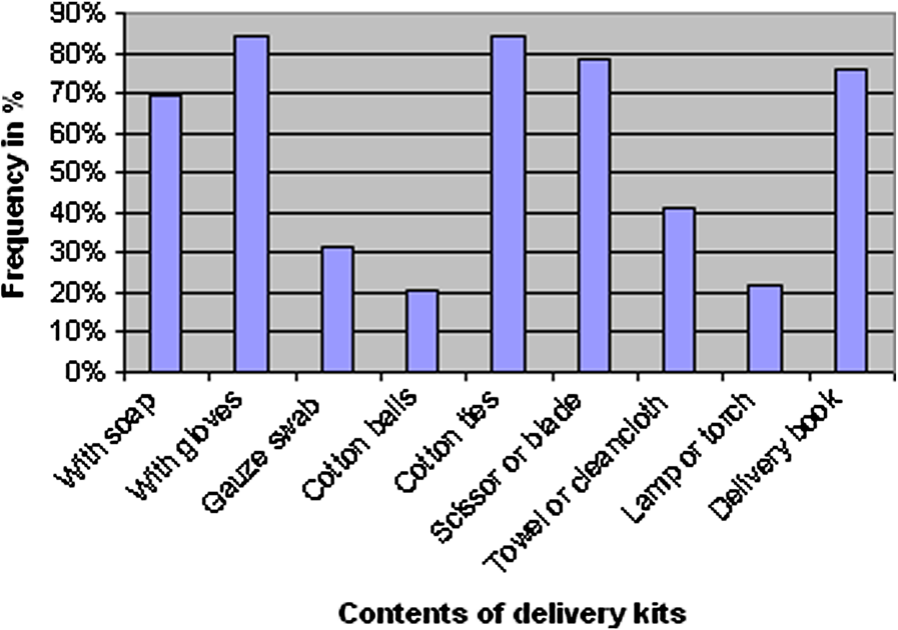
Results about the delivery kits found with the TBAs who were studied in Kigoma Rural district, Tanzania.

Of the respondents, 87.1% (n = 135) indicated to possess the delivery kits. But when such kits were inspected, it was noted that all of the respondents had the kits containing soap, gloves, cotton ties and scissors or blades. Also, of the respondents on this issue, 76.1% (n = 118) had delivery books in their kits. Only 21.6% (n = 33) had a lamp or a torch while 31.8% (n = 49) had gauze and 20.8% (n = 31) had cotton balls (Figure 1).

Procedurally, every TBA is supposed to fill in their delivery books on each delivery day the number of deliveries which s(h) has attended. When their books were reviewed, 70.6% (n = 108) out of 152 respondents were found having filled in their books; 29.4% (n = 45) of the books were blank while only 2.5% (n = 4) were missing. Finally, views were given about how best TBAs would like to be assisted in order to strengthen their effective participation and performance in safe motherhood programs. About nenety three percent ((92.9%, n = 157)) of the respondents requested to be given an opportunity to attend more training sessions on safe motherhood. They also wanted to be paid allowances and equipments for their services. The rest expressed their need for materials like uniforms and supervision by the district Council Health Management Team.

### Qualitative findings

#### Traditional medicines given to pregnant women during labour or delivery times

Using the answers to open-ended questions from interviews, it came clear that the participants could identify the medicines that were being given to pregnant women during labour or child delivery times. The commonly used and most popular one is a local herb known as *Mganasha*. This is usually given to the pregnant woman who has complications before childbirth. In particular, the leaves of this plant are pounded and then boiled in water to make juicy drink. This drink is then given to the pregnant woman to take in order to stimulate muscular contraction and accelerate her labour for immediate child delivery. If the woman after consuming this juice delays to deliver, the next step a TBA took was to insert the pounded roots of the same plant into the woman’s vagina and the belief is that by doing so the medicine works faster to facilitate contraction and help the mother deliver immediately and safely. If the *Mganasha* fails to produce the desired outcomes, the alternative medicine used is *Isoge*, another local plant/herb. It was lamented that the roots of this plant have usually been taken and given to a pregnant woman to chew and swallow the juice coming out of it which is bitter so as to accelerate muscular contraction ready for delivery. Concurrently with chewing and swallowing the juice of the roots, the pregnant woman is given part of such roots to put in her arm pit or palm – the belief being that the two actions happening together work more effectively.

#### Social beliefs attached to traditional medicines seeming to motivate communities consult TBAs before they can opt for modern care from formal health facilities

TBAs in all the study sites did not deny the point that their presence for the services they provide to community members is one of the main factors for late attendance to formal facilities by pregnant women to formal health care facilities. The participants said to have been happy with a high community trust in their services as the following statements justify:

*A child who has stayed for a long time in the birth canal before being born gets eclimpsea. This happens if the baby’s mother has not had an opportunity to take Mganasha during her labour period*. *That is why we advise them to come to us so that they can receive this medicine to prevent their babies from such a tragedy and indeed they come in numbers* (a TBA at Msimba Village in Mwandiga Division).

*Any woman who is seen by a man shortly before birth or while she is preparing to give birth at a health facility as a great chance of delivering a baby who will not respect his/her parents when such a baby becomes grows to late childhood or adulthood. Delivering at home with TBA’s assistance helps to prevent the woman from being seen by men during her labour and delivery times* (a TBA at Mtanga Village, Mwandiga Division).lso,

Reports were also given about the husbands who advise their spouses to consult TBAs first to be assured of the status of the health of their spouses and expected babies and get the desired medication before they could decide to register themselves for ANC service. It was also claimed that many women might continue consulting TBAs even after registering themselves at formal ANC clinics because of the trust they have in TBAs or little trust they have with a formal health care system. Also, the use of traditional herbal medicines before and after delivery was reported as being very common in the study communities. The reporters perceived that this revealed how the communities recognized the role of TBAs even if the women concerned appreciated the importance of formal service providers when it comes to emergencies related to pregnancy complications or after complications arising after delivery.

#### Suggestions regarding what could be done to reduce maternal deaths and improve EmoC

In the case of TBAs’ working environment the field observations made it was found that generally TBAs have been conducting their services in thatched house conditions with poor ventilation, no delivery beds and generally less hygienic surroundings. Also, some of the TBAs reported to have received delivery kits they were offered five years ago without followed-up being done by the skilled service providers from either the formal health facilities or from higher levels including the superiors from the district council level. As claimed, this would have helped them find out if there was need for replacement of such kits after a certain period of time. Among the TBAs who commented on this issue claimed that in this way it would be wrong to condemn or allege the TBAs who could be found using their own means or the old fashioned delivery kits to serve the delivering mothers.

## Discussion

Characteristically, all participants in this study were females while more than half were in the age of 50 years and above. This indicates that predominance of women and particularly women with older age in the TBA service delivery system, and this situation has been happening in other developing countries within and outside of SSA [51]. One of the possibly reasons for this to be so is that older women are more respected by their clients because of their age and the older age is perceived to reflect one’s long experience more suitability for the job than the younger ones. That is, the respondents might have regarded the older TBAs as having worked on maternal pregnancy and childbirth service issues for longer periods than the young ones. Similar observations to those made in the present study regarding the old age of majority of TBAs and other characteristics have been made elsewhere in Tanzania, for instance as indicated in the report by Twaha and colleagues entitled Twaha ALA, Muro PH, Paulo ES and George R: '*The impact of Training on knowledge and performance of TBAs in Tanga,* Tanzania: Reproductive health project, 1999’ , and the one by Leshabari that is entitled Leshabari S: '*TBAs knowledge performance and relation with the formal health system in Lindi region and the role of maternity waiting homes*, that was submitted to the Lindi Regional Health Management Team and GTZ in 2001 (both unpublished).

From the present study observations and the reviewed literature, TBAs are perceived by the public to be useful in local settings where the health care system does not satisfy all the existing needs [52,53]. Thus, lessons can be learned by health programme authorities working to improve maternal health situations in hard to reach areas by allowing the formal health care system to work in partnership with TBAs and to have a strong or effective referral system involving and supported by TBAs [52]. Thus, among other things, TBAs need to be considered when recruiting the trainees on EmOC and family health issues in general rather than leaving the nature to take its own course (i.e. instead of discarding them while they are there to exist and continue trusted by local people for their services). The training programmers and implementers need to appreciate TBAs’ role, especially after training them on various RCH matters including those addressing issues relating to referring pregnant women facing complications or with danger signs to more advanced referral centers [52]. They should also consider looking for chances that can increase men’s involvement as well and this is possible by ensuring that men are more sensitized on reproductive health planning and service seeking matters. The men are made to be self-aware about the traditional and modern methods of caring the pregnancies of their spouses, preparing for women’s childbirths and helping women to receive the necessary services during and after childbirth.

Also, the nearly 50% of the TBAs approached in this study having not acquired any kind of formal education while still attending pregnant women pregnancy and childbirth problems is a serious challenge. This is because there are possible risks when the service provider is illiterate and is uninformed of sensitive service delivery issues requiring one to have undergone some formal schooling. The investigation did not go deeper into details of looking at why such a high rate of the respondents had not gone to school and majority of them were women. However, it could be speculated or presumed that gender imbalance in access to formal education at family level contributed to this: this might have been a social-culturally rooted consequence whereby women have been more disadvantaged than men in terms of access to formal education as documented widely in the literature. First of all, the assumption is that the services given by the service providers who are lowly educated or totally uneducated might be generally of poor quality. Secondly, the observation made in the present study depicted that TBAs were generally working in the environment that was poorly equipped and this together with their low level of formal education raise another quality of service concern. Even if they were to undergo training, the lowly educated people might not have adequate ability to comprehend the key messages usually delivered through formal education including those involving written materials, leave alone the poor ability to comprehend the public health education often delivered at formal health facilities or mass media. The poorly/non educated service providers apart from not being able to read and understand sensitive issues, may shy away to seek help from the literate people and decide to maintain the status quo in their practices. The present study findings about the illiterate TBAs are consistent to the experience reported from other countries within and outside of Africa. Using specific UN indicators, it was found that majority of TBAs were illiterate and had acquired some skills by working with other experienced TBAs [51].

The experience reported about some of the clients consulting the TBAs who participated in this study daring to show their ANC cards to the TBAs is also an interesting one as it mirrors the trust such clients have had on TBAs. The clients are likely to have been of the belief that TBAs could use the information written on the cards to guide them offer the necessary care outside of the formal health clinics, although this is not necessarily true. In the training curriculum developed by the Tanzanian Ministry of Health and Social Welfare, there is a special section directing the frontline service providers to record danger signs or complications facing the pregnant women concerned. This section aims to guide any other readers of the materials concerned or anybody interested in using the MCH/ANC client’s clinic card to check for the purpose of noting if the client concerned is facing any risk conditions needing immediate or special attention by qualified service provider [45]. The present study findings also indicates that some of the respondents were unable to identify pregnancy risk conditions including the oedema of the lower limbs, abnormal lying of the baby or mal-presentations, lack of foetal movement, severe headaches, prolonged back-pain, among others. This finding is partly consistent with those reported from elsewhere in Tanzania [49] and outside Tanzania, but within SSA [54]. The take-home message here is that knowledge about danger signs during pregnancy and after delivery was still inadequate even among the TBAs who were formally trained. Therefore, so long as such providers’ services continue being recognized of their services, there is need for the health authorities concerned to continue updating the TBAs through various education and sensitization strategies.

According to the WHO [55], vaginal examination is one of the essential diagnostic actions in the assessment of the start and the progress of labour, and the number of vaginal examinations should be limited to the strictly necessary. For instance, it is stipulated that during the first stage of labour examination once every 4 hours is usually enough and if labour passes off smoothly, experienced birth attendants may limit the number of examinations to one. However, the present study findings reveal that not all the skilled maternal health service providers including the TBAs knew and observed this fact. The invasive procedures like episiotomy and amniotomy likely to cause infection were not mentioned by any one of the present study respondents. This finding is contrary to the reports obtained from the TBAs in Tanga Region, Tanzania in 1999 whereby it was noted that TBAs have been well observing such procedures frequently, according to Twaha and others. The present study did not go into details of asking why the number of vaginal examinations were reduced by some TBAs so as to establish whether or not some of the respondents were occasionally facing clients (pregnant women) who disliked being examined either at all or several times. WHO recommends that under no circumstances should women be compelled to undergo repeated or frequent vaginal examinations by a number of caregivers or students [55].

From the present study it has been observed that at least over 80% of the respondents verbally testified to have always been referring their clients to health facilities when they found it necessary to do so. While this is a good sign of the advantages of TBA training as it was observed in other places in Tanzania as noted from the report by Twaha and colleagues in 1999 mentioned above, it is still not clear whether or not the referrals reported were done earlier (before delivery) or later (after delivery). The study team did not investigate on this, hence propose further research. Efforts made by some of the respondents to this study to attend every complicated cases, are illegal since the national guidelines direct that the pregnant women with risk factors should be referred to EmoC clinics [56].

Another interesting report is that of 90% of the respondents to the present study found being aware of the HIV problem and acknowledging that transmission was possible mostly through sexual intercourses and involving skin blood contacts. However, it was sad that to hear about the TBAs having been forced by lack of gloves to assist mothers to deliver without having any means of protection; others not knowing the measures to take in order to protect themselves against the possible infections. This means, they were ready to risk themselves or their clients to HIV infections during childbirth process. This issue has to be prioritized as remedial steps since a similar problem has been found in other regions in Tanzania, for instance, in Tanga region, reported by Twaha et al., in 1999 as mentioned above. In this report it was indicated that 10% of the TBAs interviewed did not know whether there was risk of contracting HIV during their service delivery processes. Another report with similar findings has been given by Leshabari based on a study conducted in Lindi Region, also mentioned above.

As shown by the present study results, more than half of the respondents reported to have still been managing the bleeding pregnant women who contacted them irrespective of the warning they have been given during their participation at the training sessions not to do so. On one hand this is a malpractice that cannot be tolerated. On the other hand, such respondents were honest enough by confessing what they were actually doing even after attending some kind of training on EmOC issues. This is evident that not always knowledge gained is translated into the desired practice. Thus, the confessing service providers should not to be blamed for failing to comply with the guidelines considering such a challenging situation whereby they are forced to behave they find reasonable but contrary to requirements. One has to consider working in remote areas where the poor functioning of the referral system in the district is experienced and being contributed by transportation problems, other means of communication and necessary service resources required for delivering the desired type and level of the services. That is why some of the respondents expressed sympathy by deciding to assist the delivering mothers who seemed to be in urgent need for the service. It should be borne in mind that TBAs might face shame or social blames if they left their clients to die in their hands or such clients came to face more complications/problems after being allowed to go back home unassisted e.g. in situations whereby there is lack of quick and better service alternative.

Another key finding from the present study is that of the TBAs who were found to have neither been washing nor bothering to boil the instruments they were using to assist the mother in childbirth. Obviously, this would increase the risk of such providers to contract HIV infections or to transmit HIV to the delivering mothers. This is another warning to the existing health care delivery system since without attempt being made to avoid such kind of experience the debates about the rationale for involving TBAs in maternal health services including those relating to assisting mothers during delivery may not reach to an end [57].

Apart from some noted failures of TBAs to wash their working facilities, the finding from the present study showing that less than 50% of the TBAs visited could wash their hands and wear gloves before conducting a delivery service, is another policy and management challenge. Also, a little over 90% of the respondents reporting to have been doing vaginal examination by less than four times is contrary to the frequency recommended in the guidelines. These findings also indicate the substandard nature of the TBA centered services. In this situation it is difficult to give assurance beyond reasonable doubts that without additional services from skilled and personnel, the health of the mothers and newborn serviced by the unskilled/untrained TBAs would be safe from possible infections and even deaths. The experience about non-use of appropriate protective gears and following a full course of examination of the women in labour is consistent with the reports obtained from other parts of Tanzania such as Lindi region in Southern Tanzania according to Leshabari S (2001, unpublished) and Tanga Region in the northeast, according to Twaha et al., (both studies in Tanzania), as reported above.

The majority of TBAs found in this study with delivery kits that were incomplete by missing some key items while others being dirty or torn mirrors the poor environment in which TBA services were being (and might still be) delivered. For instance, conducting deliveries in thatched houses and some of the delivery kits reportedly having been issued to TBAs five years ago with no any follow-up from formal the health workers or their superiors to ensure necessary replacements is a great weakness in the service delivery environment of TBAs. Thus, without ensuring good service environments in which the TBAs carry out their duties, the training of TBAs alone could not improve the quality of care delivered to the mothers who seek TBA care. It would have been better if TBAs were received additional support from the formal sector including continued provision of essential delivery kits and supportive supervision. This might enhance their general working morale and ability to comply with the recommended EmoC standard operating procedures.

The present study is not free of limitations even if it has several strengths or advantages. It explored a very important public health topic given the fact that life begins during pregnancy, hence justifying the need for priority attention to provide necessary care to pregnant women and newborns. Understanding what goes on before, during and after childbirth in terms of health-care services is very crucial and this has remained at least in theory an important topic advocated to be accorded priority attention in the research and policy forums or agenda. On the limitation side, this study was small scale in its design, having covered only one district, a few villages and study participants, and having lacked sufficient time to explore more issues that could have helped to answer some of the key questions raised and helped the team come up with more reliable conclusion. It was mainly done for the purpose of meeting one of the academic requirements of the student (first author in this paper). This author might have bias in choosing and carrying out this study because he is one of the public health practitioners and was one of medical officers working in the public sector in Kigoma (R) district as he has been working in other regions. Also, the use of formal health workers from the district health system to collect the data could not guarantee a complete avoidance of all possible biases, both during the observations and face-to-face interviews. Thus, chances that the respondents to this study were partly influenced by the presence of formal health workers to act in particular ways while being observed and cannot be refuted with full confidence. The possibility subjective views of the health workers who participated in the data collection process cannot be denied. And as described before, the analysis presented in this paper would have informed more the reader if it showed the knowledge and practices in terms of their similarities or differences between the respondents with some different characteristics or experience. This could be possible if the originally collected data were available for more detailed analysis.

### Conclusion and policy options

The present study confirms that TBAs are key stakeholders in the health service delivery, especially in rural or remote and hard to reach settings in Tanzania as in other developing countries [53]. However, even in urban settings they can still be consulted if the existing modern health service system is perceived to lack some key quality service elements. Whether the TBAs should be allowed to operate or not is more of a research and policy question, but all in all the fact is that debates on the rationale of TBAs’ participation in maternal health services are likely to continue so long as the local communities maintain their closeness to and trust in the TBAs who carry out their business in the community settings. In light of the findings from this study, there is every reason to agree with other authors that so long as it happens that the prospects for the formal health care system to reach all pregnant women when it comes to satisfying them with childbirth services in the near future are low, one cannot deny the relevance of the point raised by the advocates for government authorities to foster synergy or partnership with TBAs. This is a true fact especially when we consider the remote settings where the professional health care givers are either lacking or inadequate [13,51]. Nevertheless, training, supervising and monitoring TBAs is imperative if the services delivered by TBAs have to be standard or improved and therefore minimising chances of malpractices. It is important to recognize the loopholes that can be (or have been) used by some of the trained TBAs to pretend providing the services which they are not eligible (or do not qualify) to deliver legally and professionally. This means, the existing laws and regulations for binding the service providers involved should be well known and strictly/effectively administered. Beside the issue of TBAs involvement, we also agree with all those who recommend effective referral systems involving TBAs [52], elimination of transport bottlenecks, improvements in the quality of services, and bringing health facilities closer to people targeted to use the services [58]. All these strategies could be achieved if the central government authorities and local government council authorities at district level do prioritize allocating necessary financial and sufficient budgets and disbursement of funds and distribution of essential materials in timely manner [59]. Achieving all of these would help to promote and improve both TBAs’ services and services delivered at formal facilities and would lead to improved physical and equitable access to MCH services, especially in hard to reach or underserved country settings.

## Competing interests

All the authors declare no competing interest in this study and have all approved the final version of this paper to be submitted for peer reviewed publication.

## Authors’ contributions

As a medical practitioner, former district medical officer for Kigoma (R) district and Singida Rural District, and a recently appointed DMO of Makambako Town Council in Njombe Region, DBV holds a Master of Public Health (MPH) degree from the Tumaini University in Tanzania; conceived this study, produced a research report as part of his MPH training at KCMC in Moshi (affiliated to Tumaini University), and participated in writing this manuscript (MS). Holding a Ph.D in Health Sciences, with training backgrounds in the areas of health economics, management, policy analysis and Finance, GMM assisted DBV in coming up with the proposed idea for research, identifying relevant literature to support it, reanalyzing and editing the qualitative and quantitative data, writing the first draft of the manuscript and revised it using comments from the co-authors of this paper and its external reviewers. MM is a MD also with Ph.D and was the supervisor of DBV throughout his thesis preparation. All authors read and approved the final manuscript.
